# Fatty Acid Metabolism in Endothelial Cell

**DOI:** 10.3390/genes13122301

**Published:** 2022-12-06

**Authors:** Bin Liu, Zhiyu Dai

**Affiliations:** 1 Division of Pulmonary, Critical Care and Sleep, University of Arizona, 475 N 5th Street, Phoenix, AZ 85004, USA; 2 Department of Internal Medicine, College of Medicine-Phoenix, University of Arizona, 475 N 5th Street, Phoenix, AZ 85004, USA; 3 Translational Cardiovascular Research Center, College of Medicine-Phoenix, University of Arizona, Phoenix, AZ 85004, USA; 4 BIO5 Institute, University of Arizona, Tucson, AZ 85724, USA; 5 Sarver Heart Center, University of Arizona, Tucson, AZ 85724, USA

**Keywords:** endothelium, vascular biology, lipid, glycolysis

## Abstract

The endothelium is a monolayer of cells lining the inner blood vessels. Endothelial cells (ECs) play indispensable roles in angiogenesis, homeostasis, and immune response under normal physiological conditions, and their dysfunction is closely associated with pathologies such as cardiovascular diseases. Abnormal EC metabolism, especially dysfunctional fatty acid (FA) metabolism, contributes to the development of many diseases including pulmonary hypertension (PH). In this review, we focus on discussing the latest advances in FA metabolism in ECs under normal and pathological conditions with an emphasis on PH. We also highlight areas of research that warrant further investigation.

## 1. Introduction

Endothelial cell (EC) metabolism has gained remarkable attention in the last few years. Accumulating evidence on glycolysis and fatty acid oxidation (FAO) in ECs has revealed that metabolism abnormalities contribute to many pathological problems including cancers and cardiovascular diseases [1,2,3,4]. In this review, we focus on discussing the latest advances in fatty acid metabolism in ECs under normal and pathological conditions such as pulmonary hypertension (PH).

## 2. Role of Endothelial Cells in Angiogenesis, Homeostasis, Stimulus/Immune Response

ECs lining the inner blood vessels are essential for the vascular system [5,6]. In adults, ECs remain quiescent for years but still retain the capacity to switch rapidly from quiescent cells to activated sprout cells to initiate new vessel formation (angiogenesis) under certain conditions such as inflammation or injury [7]. This tightly regulated process starts with the migratory tip cells and is followed by proliferating stalk cells until new vessel sprouts are created. The quiescent phalanx cells stimulated by angiogenic signals such as vascular endothelial growth factor (VEGF) then proceed to line the established vessels to form the matured blood vessels [8].

ECs occupy an important location between circulating blood and tissues, which enables its quick response to changes in the environment via releasing a host of biologically active substances including relaxing factors prostaglandin I2 (PGI2), endothelium-derived relaxing factor (EDRF), nitric oxide (NO), and endothelium-derived hyperpolarizing factor (EDHF); epoxyeicosatrienoic acids (EET); and contracting factors arachidonic acid metabolites, endothelin-1 (ET-1) and angiotensin II (Ang II) [9]. These endothelium-derived factors are maintained in a balance to control blood flow and pressure and maintain the antithrombotic and anticoagulant balance in the bloodstream, which is extremely important for homeostasis [9].

Under hormonal and chemical signal stimulation, ECs produce mediators such as nitric oxide synthase (NOS) and phospholipase A2 to modify the responses of numerous cells, including vascular smooth muscle, platelets, and leukocytes [10,11]. For instance, NO synthesized from endothelial NOS (eNOS) directly regulates blood vessel dilation by stimulating soluble guanylyl cyclase, leading to an increasing level of cyclic guanosine monophosphate (cGMP) and subsequent relaxation of vascular smooth muscle, which plays essential roles in maintaining vascular homeostasis [3,10,12].

ECs act as a natural barrier inside blood vessels and are the first to recognize microbial components in the circulation, which indicates that ECs’ recognition and response may be essential to early innate immune system activation [13]. In addition, ECs express several innate immune receptors including the Toll-like receptor (TLR), NOD-like receptors (NLR), and chemokine receptors. For instance, in response to microbial stimulation, ECs could secrete pro-inflammatory cytokine interleukin-8 (IL-8) via NOD1-dependent signaling [14].

## 3. Endothelial Cell Metabolism

EC metabolism is confined to glucose, FAs, and amino acids (AAs), the three major substrates for adenosine triphosphate (ATP) production and biomass production in ECs, which have been widely studied and summarized [1,2,15]. The following sections will discuss the role of EC metabolism (predominately focusing on fatty acid metabolism) in maintaining endothelial functions and disease pathogenesis, with a focus on pulmonary hypertension (PH).

### 3.1. Endothelial Cell Glucose and Amino Acid Metabolism

Glycolysis is the main energy resource in cultured ECs, with higher rates of glycolysis and glucose consumption [16]. ECs prefer to utilize glycolysis instead of oxidative metabolism because the ECs need to maintain reactive oxygen species (ROS) levels in control [17] and enhance the diffusion of oxygen to perivascular cells [17,18]. Additional reasons are that glycolysis produces faster kinetic ATP under pro-angiogenic signals during angiogenesis [16], which is essential for ECs’ rapid proliferation and migration [16,19]. In addition to glucose-derived carbons, ECs also utilize glutamine to sustain proliferation and vascular expansion [20,21]. Glutamine, the most abundant circulating nonessential amino acid (NEAA), can supply 30% of the tricarboxylic acid (TCA) carbons, comparable to glycolysis and FAO-derived carbon [22]. Depletion of either glutamine or arginine makes ECs vulnerable to ROS-induced damage during proliferation and migration [21].

### 3.2. Endothelial Cell Fatty Acids (FAs) Metabolism

**Figure 1:**
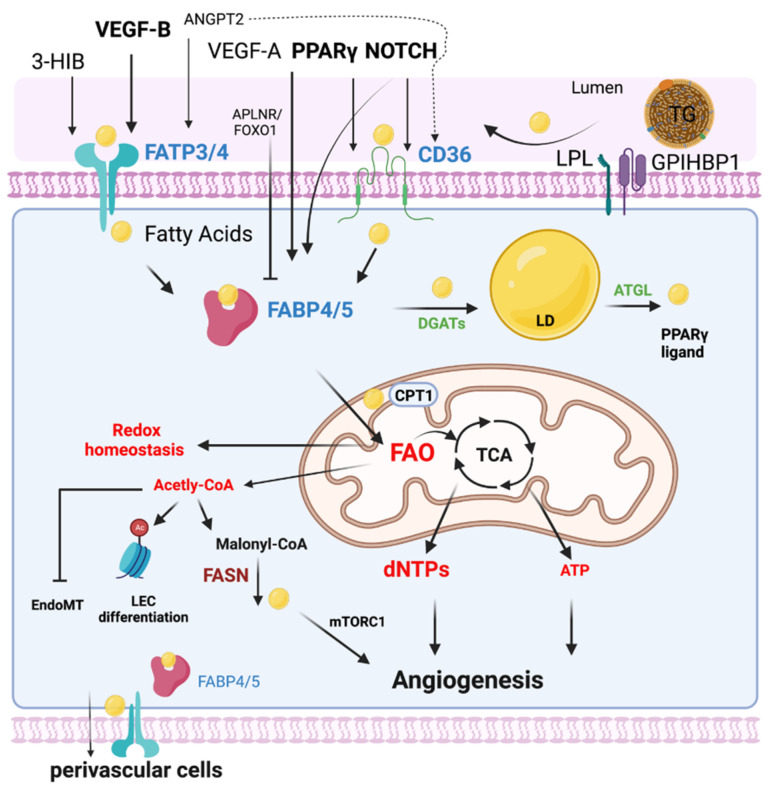
EC FA metabolism. Fatty acids (FAs) are transported in the form of triglyceride (TG)-rich lipoproteins, which are released by lipoprotein lipase (LPL) and GPIHBP1 in the luminal side of ECs. Free FAs are transported across EC membranes by fatty acid transporter protein 3 and 4 (FATP3/4) and CD36. Intracellular FAs transportation is mediated by FABP3 and FABP4. Intracellular FAs could be synthesized by FA synthase (FASN) from Malonyl-CoA and affect mTORC1 activity for angiogenesis. DAGTs and ATGL are key enzymes regulating lipid droplet (LD) storage and lipolysis. FAs are transported into mitochondria via CPT1 for FA oxidation (FAO). Quiescent ECs utilize FAO to maintain redox homeostasis. FAO is required for the generation of dNTPs for EC spouting and angiogenesis. In certain conditions such as glucose depletion, FAO produces ATP in ECs. FAO also produces acetyl-CoA for epigenetic regulation, inhibiting EndoMT. The efflux of FAs to the perivascular cells could be mediated by FABP4/5 and FATP3/4. Many key endothelial signaling including VEGF, NOTCH, and PPAR γ control EC FA transport via regulating FATPs, FABPs, and CD36.

FA metabolism involves multiple processes including FA uptake and storage, FA transport, FA oxidation, and FA synthesis. FA metabolism is vitally important to sustain the function of organs such as the heart, skeletal muscle, and adipose tissue [15]. Heart and skeletal muscles utilize FAs as their top source for ATP production and therefore require an efficient supply system of FAs [23]. Uptake and transport of FAs by ECs are extremely important to numerous cellular processes, including membrane synthesis, intracellular signal transduction, ATP generation, protein posttranslational modifications, and metabolic gene transcriptional regulation in these high energy-demand organs [24]. This process requires the transport of FA across the EC barrier into the perivascular cells [25] (Figure 1). In addition, ECs can metabolize and synthesize FAs for maintaining vascular homeostasis.

#### 3.2.1. Endothelial Cell Fatty Acid Uptake and Transport

In mammals, lipids circulate as nonesterified free fatty acids (NEFAs) in the blood including medium-chain (with 6–12 carbons) and short-chain fatty acids (SCFAs) (with 6 carbons), but mostly (≈90%) as esterified FA, including long chain fatty acid (LCFAs) (with 12–20 carbons). The LCFAs are transported in the bloodstream in the form of triglyceride (TG)-rich lipoproteins, phospholipids, and cholesteryl esters in lipoproteins [26], while uptake LCFAs requires transfer from the circulation across the EC barrier, a process coordinated by several membrane proteins including lipoprotein lipase (LPL) and GPIHBP1 [27], CD36 [28], fatty acid transport proteins (FATPs) [29], and fatty acid-binding proteins (FABPs) [30,31,32]. The major fatty acid transporter including CD36, FATPs, and FABPs will be summarized in the following sections (Figure 1).

##### CD36

CD36 is the best-characterized FA transporter [33]. CD36 is expressed in platelets, monocytes, ECs, and parenchymal cells in white adipose tissue (WAT), brown adipose tissue (BAT), heart muscle, and skeletal muscle [34]. Besides its important biological roles in binding LCFAs and facilitating their uptake into cells [28], CD36 also recognizes lipoproteins, bacterial lipids, and nonlipid ligands (e.g.,thrombospondin-1) [34,35,36]. CD36 plays a very important role in FA import into cells and is involved in a series of cell signaling processes including apoptotic cell uptake, cellular adhesion, and angiogenesis [37,38,39,40]. EC-Cd36–KO mice had reduced uptake of FA in the heart, skeletal muscle, and BAT, suggesting that endothelial CD36 plays a critical role in FA delivery into parenchymal tissues [41]. ECs utilize FAs to accelerate their migration and invasion for new vessel formation following vascular injury in a CD36-dependent manner likely through AMPK signaling [42,43]. It has also been reported that EC-specific knockout of CD36 in mice had promoted glucose clearance compared with controls when fed with multiple diets and female EC CD36/LDLR-deficient mice have reduced atherosclerosis [44]. Other studies showed that intestinal lymphatic endothelial cells (LECs) CD36 regulates lymphatic integrity and optimizes lipid transport. Silencing CD36 in cultured LECs suppresses cell respiration via reducing VEGF-C-mediated VEGFR2/AKT phosphorylation and destabilizing VE–cadherin junctions [45]. Moreover, mice with specific deletion lymphatic Cd36 display slower transport of absorbed lipids, more permeable mesenteric lymphatics, accumulation of inflamed visceral fat, and impaired glucose disposal [45].

##### FATPs

FATPs are trans-membrane-spanning proteins, which allow and enhance the uptake of LCFA into cells [32]. Compared with other FATPs, FATP3 is specifically expressed in the vasculature in muscular tissues [23], whereas FATP4 is expressed in endothelium and muscle and WAT [23]. Previous studies demonstrated that VEGF-B specifically controlled endothelial uptake of FA via transcriptional regulation of vascular FATP3 and FATP4 in muscle, heart, and BAT through VEGF receptor 1 and neuropilin 1 [23]. Dr. Arany’s group later identified 3-hydroxyisobutyrate (3-HIB), a catabolic intermediate of the branched-chain amino acid (BCAA) valine, promotes trans-endothelial fatty acid transport through FATP3 and FATP4, stimulates muscle fatty acid uptake in vivo and promotes lipid accumulation in muscle, leading to insulin resistance in mice [46]. Another study found that Angpt2 activated integrin α5β1 signaling in the endothelium and triggered fatty acid transport via CD36 and FATP3 into subcutaneous adipose tissue (SAT) [47]. Surprisingly, mitochondrial ATP has been demonstrated to control endothelial FA uptake via endoplasmic reticulum (ER)-FATP4 through its ATP-dependent acyl-CoA synthetase activity [48].

##### The FABPs

The FABPs belong to a family that consists of at least 13 intracellular FA-handling proteins [32]. The FABPs can directly bind free LCFA and facilitate the delivery of LCFA to different intracellular sites for oxidation. Their expression pattern is tissue-specific [32]. Among the FABPs, FABP4, also known as A-FABP and aP2, is widely studied in ECs compared to other FABPs [49,50]. FABP5, also known as E-FABP and mal1, is found in the ECs of the microvasculature of the placenta, heart, skeletal muscle, small intestine, and lung [30,51]. FABP3 or h-FABP is found in the heart microvessels and aortic endothelial cells [38].

FABP4 and FABP5 share a 55% similarity of amino acid sequence and have a primarily microvascular expression pattern in both microvascular and large blood vessel ECs [51,52]. FABP4 is regulated by VEGF and contributes to EC proliferation, migration, and angiogenesis [50]. FABP4 exhibits a pro-angiogenic role in ECs by modulating the stem cell factor/c-kit pathway [53]. Other studies showed that exogenous FABP4 treatment induced EC dysfunction by impairing insulin-dependent eNOS activation and NO production through suppression of IRS1 and Akt signaling [54]. In addition, human coronary artery endothelial cells (HCAECs) derived FABP4 could increase inflammatory cytokines, and proliferation and adhesion-related molecules to promote human coronary smooth muscle cells proliferation and migration, which reveals the role of FABP4 in neointima formation after vascular injury [55]. Consistent with these studies, FABP4-specific inhibitor, BMS309403, exhibits therapeutic potential in the treatment of EC dysfunction-associated diseases including atherosclerosis and diabetes [56,57]. A recent study also showed that tumor endothelial cells (TECs)-derived FABP4 promoted lipid droplet formation in cancer cells and protected them from oxidative stress-induced ferroptosis [58].

There is a redundant function between FABP4 and FABP5, as Fabp4 and Fabp5 (Fabp4/5 double-knockout (DKO) mice) exhibited a dramatic phenotype related to protection against obesity, insulin resistance, atherosclerosis, and fatty liver disease compared to Fabp4-/- or Fabp5-/- mice [59,60]. Interestingly, even though FABP5 shares a similar expression pattern with FABP4 in microvascular beds of several tissues, and functions the same as FABP4—significantly enhancing EC proliferation, chemotactic migration, and angiogenic sprouting—FABP5 promotes apoptotic EC death under certain conditions, which is opposite from the anti-apoptotic effects of FABP4 [61]. FABP5 has been demonstrated to mediate the uptake of exogenous docosahexaenoic acid (DHA) in brain ECs and contributes to cognitive function [62].

Both FABP4 and FABP5 are co-expressed in capillary ECs across various organs, including the heart, and skeletal muscle. Key endothelial signaling has been shown to control FABP4 and/or 5 expressions for transendothelial FA transport. For example, endothelial PPARγ promotes FA uptake via CD36 and FABP4 [63]. EC Notch signaling induces FABP4/5 and CD36 to facilitate FA transport in the heart [64]. A recent study demonstrated that EC APLN/APLNR signaling suppressed the transcription factor Forkhead box protein O1 (FOXO1) in the endothelium and inhibited endothelial FABP4 expression, which limits the excess FA transport and accumulation in the tissue [65].

#### 3.2.2. Endothelial Fatty Acid Storage and Lipolysis

Lipid droplets are present in most eukaryotic cells including ECs for cytosolic organelles fat storage [66]. Lipid droplets could buffer excess lipids to protect ECs from lipotoxicity and release them later according to cellular consumption [66]. Lipid droplets are strictly regulated under cellular needs and environmental signals, which is tightly balanced by LD biogenesis and lipolysis.

Large blood vessel ECs can form and degrade lipid droplets in response to changing levels of TG, in vivo. Combined with the pharmacological inhibition of DGAT1 and DGAT2, the Sessa group demonstrated that DGATs are essential for TG synthesis in ECs with a greater dependency on DGAT1 than on DGAT2 [67]. Inhibition of DGAT1 but not DGAT2 was sufficient to promote ER stress induced by exogenous oleic acid treatment, suggesting that the capability of EC to form lipid droplets is critical for the prevention of lipotoxicity [67].

Lipolysis is determined by the direct activation of LD-associated lipases, such as adipose triglyceride lipase (ATGL), monoglyceride lipase (MGL), and hormone-sensitive lipase (HSL) [67,68]. They further showed that ATGL inhibition but not HSL or MGL inhibition delayed lipid droplet degradation in ECs [67]. ATGL-deficient mice present severe micro- and macrovascular endothelial dysfunction [69], including impairment of vasodilatory, and reduction of eNOS/cGMP signaling. In human aortic endothelial cells (HAEC), silencing *ATGL* decreased the efficiency of stimulus-induced arachidonic acid (AA) release and prostacyclin secretion [70]. Adipose tissue microvascular endothelial cells (aMVECs) ATGL, but not HSL, plays an essential role in regulating adipose tissue lipid uptake through secreting PPARγ ligands [71].

#### 3.2.3. Endothelial Cell Fatty Acid Oxidation

Glycolysis is the predominant bioenergetic pathway for ECs, as ECs generate 85% of their ATP via glycolysis [16]. Fatty acid oxidation (FAO) generates only 5% of the total amount of ATP in naive ECs [16]. Some studies showed that FAO compensates for ATP production up to 40% in ECs when glucose is removed or when lipid is provided in the presence of the AMPK activator AICAR [72]. Later studies showed that FAO-derived carbons sustain the TCA cycle to generate the precursor of nucleotides, aspartate, for deoxyribonucleotide (dNTP) synthesis during EC proliferation [22]. Silencing carnitine palmitoyltransferase 1 (*CPT1A),* the rate-limiting enzyme for FAO, disrupts EC sprouting in vitro and in vivo due to a decrease in the dNTP pool [22]. Surprisingly, quiescent ECs express a higher level of FAO genes to sustain the TCA cycle for redox homeostasis through NADPH regeneration. EC *CPT1A* deficiency in mice promotes EC dysfunction by increasing oxidative stress [73]. Indeed, inhibition of FAO via hyperoxia/air recovery leads to lung EC apoptosis and lung injury in mice [74]. However, other studies demonstrated that increased FAO in aortic ECs promotes the generation of superoxide, and inactivates prostacycline synthase and eNOS in the context of insulin resistance [75]. Moreover, lymphatic ECs proliferation during lymphatic development also requires FAO to produce dNTPs [76,77]. Interestingly, FAO-derived production of acetyl-CoA promotes histone acetylation of PROX1-target (lymphatic) genes and lymphatic EC differentiation [76]. Moreover, silencing EC FAO via carnitine palmitoyltransferase II deletion (Cpt2-KO) in mice causes augmented embryonic endothelial-to-mesenchymal transition (EndoMT), thickening cardiac valves and increased vascular permeability in these mice [78]. Taken together, ECs use FAO for redox homeostasis, DNA synthesis, acetyl-CoA generation/epigenetic regulation, and ATP production.

Although fatty acid synthesis is generally low in healthy cells, in vitro pharmacological inhibition of Fatty acid synthase (FASN), which converts malonyl-CoA to free FA, reduces EC proliferation and downregulation of vascular endothelial growth factor receptor 2 (VEGFR2) [79]. Endothelial-specific deletion of FASN impaired angiogenesis in mice, while pharmacological FASN blockade with a low dose of orlistat reduced pathological ocular neovascularization. The impairment of angiogenesis is not due to energy stress, redox imbalance, or palmitate loss, but malonylation modification of mTOR Complex 1 (mTORC1) [80]. In addition, the inactivation of endothelial FASN (Tie2Cre mediated FASN knockout) decreases eNOS palmitoylation (bioavailability) and increases vascular permeability in mice [81]. The role of lipogenesis in vascular physiopathology needs to be further investigated.

### 3.3. Fatty Acid Metabolism in Pulmonary Hypertension

**Figure 2:**
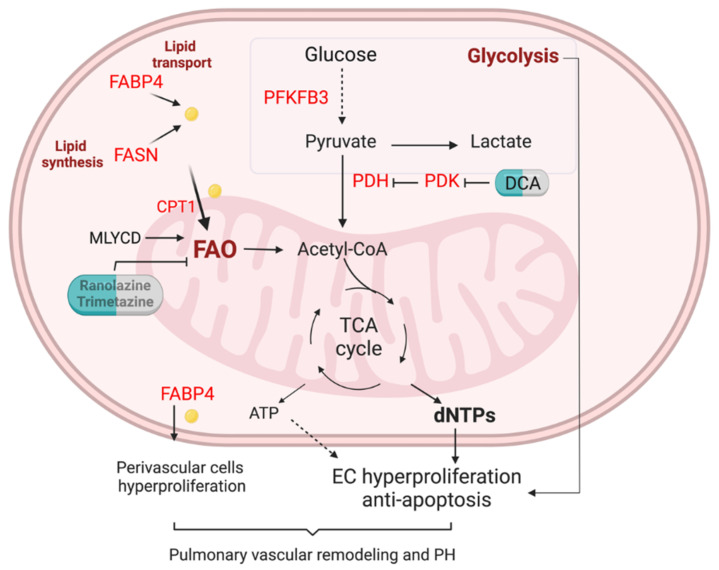
FA metabolism in the lung EC of pulmonary hypertension. Enhanced glycolysis is evident in PAH ECs. Lipid transport via FABP4, lipid synthesis via fatty acid synthase (FASN), and fatty acid oxidation (FAO) are unregulated in pulmonary arterials of PAH patients. Elevation of FA could increase mitochondria FAO, contributing to the generation of dNTPs and ATP. Enhanced FAO and glycolysis lead to PAH EC hyperproliferation and anti-apoptotic phenotypes. Upregulation of FAPB4 could also increase FA efflux to perivascular cells such as pulmonary arterial smooth muscle cells and fibroblasts, and promote pulmonary vascular remodeling and PH.

Given multiple molecules and their complex regulation network involved in FA metabolism in ECs, any abnormality in these regulations will lead to a pathological state and even develop into a disease. Here, we will only discuss the role of FA metabolism and its related pathway in pulmonary hypertension (PH) (Figure 2).

Pulmonary arterial hypertension (PAH), or group 1 PH, is an incurable lung disease, characterized by increased resistance in the pulmonary vascular system, which will finally lead to elevated resting pulmonary artery pressure and end by right ventricular failure [82]. Accumulating evidence has demonstrated that EC dysfunction plays a crucial role in the initiation and progression of PAH, manifested by increased susceptibility to injury and enhanced proliferation, contributing to the formation of plexiform lesions [83,84,85,86]. Indeed, most PAH mutation-causing genes are mainly expressed in the lung ECs based on the human lung single-cell RNA sequencing analysis [87].

To date, increasing evidence indicates that EC metabolism dysfunction is closely associated with the pathogenesis of PAH [88,89,90]. The major observed metabolic alteration of EC metabolic change in PAH has increased glycolysis [88]. Glucose metabolic activities were higher in the lungs of PAH patients than in healthy individuals determined by in vivo fluorodeoxyglucose (FDG)-positron emission tomography (PET) scans [91,92]. Healthy ECs generate 85% of their energy from glycolysis. The glycolytic rate of PAH ECs is even greater compared to healthy ECs [91,92], suggesting that PAH ECs exhibit a further shift to aerobic glycolysis. Pulmonary vascular ECs sustain the proliferative and anti-apoptotic phenotypes depending on enhanced glycolysis. Mice with EC-specific deletion of PFKFB3, a key regulator of glycolysis, slowed PH development [93].

FA metabolism abnormality has been observed in PH. Metabolites profiling of the plasma from patients with PAH and healthy controls showed the dysregulated metabolic pathways. Metabolites representing lipid metabolism and fatty acid were closely associated with PAH [94]. Circulating levels of FABP4 were significantly elevated in PAH patients identified by plasma proteome profiling [94]. Lipid deposition is increased in the lungs of PH patients compared to control lungs [95]. FASN was upregulated in hypoxic pulmonary arterial smooth muscle cells (PASMCs) and pulmonary arterial ECs (PAECs), and monocrotaline (MCT)-treated rats (a widely used PH model) [79,96]. FASN inhibition using the C75 compound decreased right ventricular pressure, right heart hypertrophy, pulmonary vascular remodeling, and endothelial dysfunction in MCT-exposed rats [96] and hypoxic mice [97].

Previous studies showed that non-esterified free fatty acids and acylcarnitines in the circulation are upregulated in patients with PAH compared to healthy controls [98,99]. Using a combination of high-throughput liquid-and-gas-chromatography-based mass spectrometry analysis on human PAH lung, Zhao et al. observed that there were increased long- and medium-chain free fatty acid products accumulated in PAH tissues compared to control lung, a reflection of mitochondria β-oxidation [100]. They also demonstrated an increase of omega-oxidation in fatty acids and upregulation of lipid oxidation in the lung of PAH [100]. Recent studies also demonstrated the upregulation of FA uptake, processing, and β-oxidation-related genes in the laser-dissected pulmonary arteries from idiopathic PAH (IPAH) patients [101]. Our unpublished data showed that PAECs isolated from IPAH patients exhibited increased exogenous FAO compared to PAECs from failed donors using Seahorse XF Palmitate Oxidation Stress assay, suggesting upregulation of FAO in PAH EC. Other evidence showed that inhibition of FAO via genetic deletion of malonyl-CoA decarboxylase (MYLCD) in the whole body protected animal models from developing PH in mice [102]. Genetic knockdown of Cpt1a in mice or pharmacological inhibition (Oxfenicine) of Cpt1b in rats attenuated pulmonary vascular remodeling and PH [101].

In contrast, other studies showed a decrease in carnitine acyltransferase (CrAT), indicative of the reduction in FAO, in the endothelium of congenital heart disease and the PH lamb model [103]. Metabolomic analysis of bone morphogenetic protein receptor type 2 (BMPR2) mutations, which account for 80% of heritable PAH, in human pulmonary EC reveals the downregulation of the carnitine and FAO pathway [104].

PAH patients mostly die because of right heart failure. However, the role of FAO in RV failure is also controversial. Recent studies have demonstrated that intracellular lipid accumulation and the reduction of FAO are features of right heart failure secondary to PAH [98,99]. Human right ventricular (RV) long-chain FAs, myocardial triglyceride content, and ceramide were increased and long-chain acylcarnitines were markedly reduced in PAH versus controls [98]. Activation of FAO using PPAR-γ agonist pioglitazone or metformin or L-carnitine decreased lipid accumulation and improved right heart function in PH animal models [99,105,106]. However, another group demonstrated that partial inhibition of FAO using Trimetazidine restored pyruvate dehydrogenase (PDH) activity and glucose oxidation, and improved RV function in pulmonary artery banding rats [107] via manipulating Randle’s cycle, for example, inhibiting FAO increases glucose oxidation, or vice versa [107].

Taken together, the role of endothelial FA metabolism in the pathogenesis of PAH and RV failure is complicated and needed for further investigation.

## 4. Therapeutic Opportunities of FA Metabolism in PH

The current PAH therapies do not prevent the progression or cure the disease. The 5-year mortality of PAH is still as high as 40% [108]. The metabolic intervention via targeting glycolysis and FAO in PH patients has been explored in clinical trials. Pyruvate dehydrogenase kinase (PDK), a negative regulator of the mitochondrial enzyme pyruvate dehydrogenase (PDH), was upregulated in the wall of muscularized pulmonary arteries and remodeled RV from PAH patients compared to non-PAH donors, indicating the inhibition of glucose oxidation and upregulation of glycolysis in the pulmonary arteries and RV of PAH patients [109,110,111]. Perfusion of PDK inhibitor dichloroacetate (DCA) increased PDH activity and increased mitochondrial respiration in human PAH lung ex vivo [109]. DCA administration in 4 months to IPAH patients led to a reduction in mean PA pressure and pulmonary vascular resistance and improvement in functional capacity [109], suggesting PDK is a druggable target for PAH treatment.

Multiple FAO inhibitor trials are completed or on the way [112]. Ranolazine has been demonstrated to reduce calcium overload through inhibition of FAO and the in-ward late sodium current (I_Na_) [113]. FAO inhibition using ranolazine treatment has been tested in a small number of PAH patients (*n* = 10) for 3 months [114]. Ranolazine treatment reduced RV size, and improved RV function, but did not affect hemodynamic parameters [114]. A recent completed double-blind, randomized, placebo-controlled Ranolazine trials (*n* = 9 Ranolazine, *n* = 6 placebo) showed that ranolazine treatment improved RV ejection fraction but not 6-min wall distance (6MWD), N-terminal pro-brain natriuretic peptide, or quality of life measures [115]. Another Ranolazine trials (NCT02133352) and Trimetazidine trial (NCT02102672) are still ongoing.

There is accumulated evidence that PAH is associated with systemic metabolic disorders including metabolic syndrome and insulin resistance [116]. Metformin is a widely used oral anti-diabetic drug that improves insulin sensitivity, increases fatty acid oxidation, and reduces oxidative stress [117]. The therapeutic role of metformin is investigated in both PAH and PH associated with congenital heart defects (PH-CHD) [118,119]. In a single-center, open-label 8-week phase II trial of metformin in PAH trial (*n* = 20), Metformin did not change the 6MWD but did significantly improve RV fractional area change and reduced RV triglyceride content [118]. In another study, Metformin was added to the Bosentan therapy in patients with PH-CHD. The improvements in 6MWD and pulmonary vascular resistance were significantly greater in PH-CHD patients treated with Metformin plus Bosentan therapy than in those who received Bosentan only [119].

The understanding of the metabolic mechanisms including FA metabolism in the pathogenesis of PAH is critical in the development of novel therapeutic strategies. The current understanding of EC FA metabolism remains elusive. The observation of FAO in the lung ECs and RV is inconsistent based on the studies from different groups (describe in Section 3.3, which might be due to the differences in the experimental models and approaches in individual laboratories. Further studies should employ comprehensive approaches such as single-cell omics, spatial transcriptomics, proteomes, and metabolomics to understand the metabolic alteration in PAH samples. To demonstrate the proof-of-concept of FA dysfunction theories in PH, genetic manipulation of a certain gene in the animal models are superior compared to pharmacological targeting of the protein of interest due to the potential off-target effect of chemical compounds [120]. In addition, severe PH mice models, for example, *Egln1^Tie2Cre^* [83,121] and IL6-Tg [122] mice, might be used as the PH phenotype of hypoxic mice model is mild which does not reproduce the human pathology [123].

In our perspective, EC FA transport signaling represents a novel therapeutic approach for the treatment of PAH. Limiting the EC FA transport will reduce FAO/dNTP axis and attenuate EC hyperproliferation in the lung ECs and pulmonary vascular remodeling and PH. On the other aspect, inhibition of EC FA uptake could potentially reduce the lipid deposit and lipotoxicity in the lung and RV cardiomyotes, and improve RV function. In conclusion, improved understanding of the EC FA metabolism responsible for the initiation and progression of PAH will facilitate the development of effective treatments for PAH.

## Data Availability

Not applicable.
